# Comment on: “Hepatitis C Virus in Arab World: A State of Concern”

**DOI:** 10.1100/2012/534173

**Published:** 2012-09-27

**Authors:** Gasim I. Gasim

**Affiliations:** Department of Medicine, College of Medicine, Qassim University, P.O. Box 6655, Buraidah 51452, Saudi Arabia

Dear Sir,

I have read the article by Daw and Dau with great interest and I have to congratulate the authors. However, the most recent prevalence of hepatitis C among hemodialysis patients in Sudan is stated to be 8.5% [1]. This puts Sudan among the lowest Arab countries in terms of prevalence of hepatitis C among hemodialysis, a thing that might change your conclusions on speaking about the lowest prevalence among Arab countries.

Authors mentioned that the risk factors are associated with hepatitis C virus contraction. Gasim et al. found that the duration of hemodialysis is significantly associated with HCV contraction [1] and this could be explained by the fact that this HCV infection is a sort of a nosocomial one.

Among the Japanese, recent data discussing the seroprevalence of HCV antibodies found it to vary between 8.5 and 12.5% [2], a thing which might be different from what the authors have mentioned when they spoke about a prevalence of 1.2%. 

Furthermore, there is a concern that authors referred to the paper titled *Epidemiology of Hepatitis B and Hepatitis C Virus Infections among Hemodialysis Patients in Khartoum, Sudan* [1] wrongly in paragraph 13 lines 5 and 6 that it showed the main hepatitis C virus genotype to be 4 with the subtypes 4e, 4c, and 4d while the fact is that the study showed the epidemiology of hepatitis C among hemodialysis, patients without addressing the matter of genotypes whereas Mudawi et al. 2007 described the genotypes, among patients with hepatosplenic schistosomiasis [3]. Thus, Mudawi et al. is the suitable reference rather than the mentioned one, and you might cite Gasim et al. 2012 in the lines speaking about seroprevalence. 

 I'm afraid that the references [46–48] were mismatched between the citation in the text and the references list; I would be very much grateful if these were checked.
